# Editorial: Exosomal biomarkers: roles in diagnostics and therapeutics, Volume II

**DOI:** 10.3389/fmolb.2025.1547186

**Published:** 2025-01-30

**Authors:** Dwijendra K. Gupta

**Affiliations:** Department of Biochemistry, Allahabad University, Allahabad, India

**Keywords:** exosomal biomarkers, theranostic, non-invasive therapeutics, EV biogenesis, salivary EV subsets, exosome bibliometrics

Extracellular vesicles (EVs) or Exosomes, are membrane-enclosed particles secreted by a variety of cell types and encapsulate a diverse range of molecules, including proteins, nucleic acids, lipids, metabolites, and even organelles derived from their parental cells. Further, the composition of EVs can reflect on the health and disease status of the donor cell. The crucial mediatory role of exosomes in intercellular communication, gene expression, signaling pathways, and cellular behavior is well documented. The proposed Research Topic is a collection of original research, reviews and mini-reviews, to help us understand role of exosomal dynamics towards cost-effective diagnostics, non-invasive biopsy, novel therapeutic strategies and solutions toward infertility treatments and neurodegenerative diseases.

Poorly understood at present are the intricate processes involved in EV biogenesis by donor cells and subsequent uptake by recipient cells. Thus, a thorough understanding of EV biogenesis is crucial for the development of EV-based therapeutics. This aspect is aptly reviewed by Yu et al. who have extensively discussed the fundamental mechanisms involved in EV biogenesis and delivery and also address on the potential of basic mechanistic research in developing novel diagnostic strategies and therapeutic applications.

In chronic inflammatory lung diseases and lung cancer, distinctive phenotypic changes in macrophage are concomitant with M1-predominant macrophages promoting inflammatory damage and M2-predominant macrophages fostering cancer progression. Exosomes released by reshaped macrophages play a dual role: firstly, by reshaping anti-inflammatory macrophages, inhibiting pro-inflammatory macrophages, and secondly, by alleviating inflammatory damage. Hence, a thorough understanding of pathogenesis of lung cancer and search for novel therapeutic approaches becomes necessary. Kang et al. in their review discuss efficient and targeted exosome-based therapies that may emerge as an attractive option for the treatment of pulmonary diseases. In a systematic review article, Zhong et al. present a comprehensive review of exosome research in lung cancer and its progress through bibliometric analytics of exosomes from lung cancer patients during the period of 2004–2023.

In a study on Extracellular Vesicles (EVs) isolated from U-937 cells and characterized by DLS and immunoblotting, Rathi et al. have inquired whether EVs can modulate NADPH Oxidases (NOX4 and NOX2), that have a key role in production of reactive oxygen species (ROS), in monocytes and macrophages. Their results demonstrated that the exogenous addition of differentiation agents (Phorbol 12-myristate 13-acetate (PMA) or ascorbic acid) or the supplementation of EVs used in their study did not cause any stress leading to alterations in cell proliferation and viability. Interestingly, the cells co-cultured with EVs for 72 h, the transformation of monocytes into macrophages exhibited strong suppression of NOX4 and NOX2. This provided evidence for NOX regulation by EVs in addition to its role as an antioxidant source in conformity with previous studies that point out to the role of EVs in therapeutics.

Can exosomes also play a role to help better understand the body-mind perspectives in health? The trigger of the immune system *via* activation of monocyte-derived exosomes also suggest a role for the gut-brain immune axis as an attractive theranostic target. By inducing spontaneous changes in microRNAs in the brain endothelial cells, these exosomes cause an acute inflammatory response with physiological and psychological sequelae, like anxiety and depression. Rajan et al. have explored the role of exosomes in stress models of anxiety and depression,prospecting theranostic potential of Psycho-Neuro-Endocrinological Perturbation into the gut-brain immune axis and downstream cardiovascular sequelae.

A number of strategies, like. mammograms, Fine-Needle Aspiration Cytology and biomarker tracking, are currently in use for breast cancer that affects the women globally with high rates of morbidity and mortality. Early diagnosis of the disease still remains a challenge as no effective strategy has yet been evolved to facilitate rapid and precision oriented screening of breast cancer, Presence of exosomes in body fluids could be an attractive candidate for the rapid screening of breast cancer. In this context, exosomal microRNAs- that play a significant role in modifying the tumour microenvironment of breast cancers, in the proliferation, invasion and metastasis of breast cancer appear to be a choice of non-invasive diagnostic tool. In a search for novel strategies for effective treatment, an excellent review by Gulati et al. summarizes the possible role of ExomiRs to serve as candidate biomarkers for facilitating rapid screening and triaging of breast cancer patients for therapeutic intervention.

As a follow up of an earlier investigation on human salivary enzymatic activity of Dipeptidyl Peptidase IV(DPP IV) pointing out to existence of two subsets of exosomes, Ogawa et al. developed a simple method to prepare two distinctive EVs, termed EV-I 20 k-ppt and EV-II 100 k-ppt, from just 1 mL of human WS which is based on a combination of size-exclusion chromatography of improved condition and sequential centrifugation. Based on proteomics analytics and immune-precipitation studies they further characterized the two fractions, EV-I 20 k-ppt and EV-II 100 k-ppt,that were not only distinctive with regard to size but also different with regard to constitutive proteins. While the Fraction EV I 20 k-ppt contained aminopeptidase N (APN), mucin 1, ezrin, and Annexin A1,the characteristic proteins of EV-II 100 k-ppt fraction comprised DPP IV and CD9, programmed cell death six-interacting protein, and tumor susceptibility gene 101. Interestingly, Ogawa et al. also found these two salivary exosomal fractions distinctive with regard to their binding to coronavirus spike proteins. Whereas EV-II 100 k-ppt, significantly bound to the spike protein of Middle East respiratory syndrome coronavirus (MERS-CoV), EV-I 20 k ppt, did not bind. What pathophysiological roles these two types of salivary EVs with distinctive features and functions play in the oral cavity and gastrointestinal tract remains to be investigated.

Since the course of pregnancy is immune-programmed through modulation of macrophage (M1/M2) differentiation, Extracellular Vesicles (EVs) in maternal blood could play a role as pregnancy predictor of normal term birth (TB) and preterm birth (PB). Huang et al. in their original research article investigated whether urinary EVs (UEVs) could serve as a non-invasive mechanistic biomarker in predicting PB. In their investigation on first-trimester UEVs carrying M1 messengers with altered immune programming, the group has attempted to discern their correlation to subsequent PB. This research group observed a significant elevation in the particles of UEVs bearing characteristic exosome markers (CD9/CD63/CD81/Syntenin) during first-trimester of pregnancy in comparison with that in non-pregnant samples. Interestingly, UEVs from TB exerted a significant suppression of M1 differentiation (iNOS expression) and Th17 differentiation (RORrT expression) compared to those of PB. Their pioneering study provided critical evidence for the early detection of altered M1 and Th17 responses within UEVs as a predictor of PB.

